# Antifungal exposure in *Scedosporium* septic arthritis: synovial penetration of voriconazole and terbinafine and plasma concentrations of olorofim

**DOI:** 10.1128/aac.00415-26

**Published:** 2026-07-13

**Authors:** Chemsa Le Cœur, Olivier Le Tilly, Adélaïde Chesnay, François Darrouzain, Roger J. M. Brüggemann, Damien Richard, Fanny Lanternier, Dea Garcia-Hermoso, Adrien Lemaignen, Marion Lacasse

**Affiliations:** 1 Service de Maladies Infectieuses et Tropicales, Hôpital Bretonneau, CHRU de Tours 26928 , Tours, France; 2 Service de Pharmacologie Médicale, CHRU de Tours 26928 , Tours, France; 3 Université de Tours, Inserm, UMR1327 ISCHEMIA, Membrane Signalling and Inflammation in Reperfusion Injuries, Tours, France; 4 Service de Parasitologie–Mycologie–Médecine Tropicale, CHRU de Tours 26928 , Tours, France; 5 Department of Pharmacy, Pharmacology and Toxicology, Radboud University Medical Centre 6034, Nijmegen, the Netherlands; 6 Radboud Institute for Medical Innovation, Radboud University Medical Centre 6034, Nijmegen, the Netherlands; 7 Service de Pharmacologie Médicale, CHU Gabriel-Montpied 55448 , Clermont-Ferrand, France; 8 Service de Maladies Infectieuses et Tropicales, Hôpital Necker, Assistance Publique-Hôpitaux de Paris (AP-HP), Paris, France; 9 Département de Mycologie, Université Paris Cité, Centre National de Référence des Mycoses invasives et Antifongiques, Institut Pasteur 27058, Paris, France; 10 xEA 7505, Education, Ethique, Santé, Université de Tours 27092, Tours, France; University Children's Hospital Münster, Münster, Germany

**Keywords:** *Scedosporium*, diffusion, penetration, synovial fluid, voriconazole, terbinafine, olorofim

## LETTER

*Scedosporium ellipsoideum*, phylogenetically close to *Scedosporium boydii*, is a rare cause of invasive fungal disease with osteoarticular tropism (1). Current expert recommendations support the use of systemic antifungal therapy combined with surgical debridement when feasible (2); however, data on antifungal penetration into inflammatory synovial fluid remain scarce.

We report a 73-year-old immunocompromised man with profound acquired B-cell and CD4+ T-cell lymphopenia and severe IgG hypogammaglobulinemia, who developed *S. ellipsoideum* fungemia complicated by multiple articular localizations, including septic arthritis of the native left knee joint. Diagnosis was confirmed by positron emission tomography–computed tomography (PET-CT) imaging and joint aspiration. *S. ellipsoideum* was repeatedly isolated from blood cultures, synovial fluid, and bone biopsy. MICs (EUCAST) were as follows: amphotericin B 2.0 mg/L, voriconazole 0.5 mg/L, micafungin 0.25 mg/L, terbinafine >8 mg/L, and olorofim 0.015 mg/L. The patient had normal serum albumin and a CYP2C19 *2/*17 genotype (intermediate metabolizer for voriconazole).

Initial therapy with voriconazole (200 mg twice daily, increased to 300 mg) was followed by arthrotomy after 3 months due to progressive knee disease. Ongoing infection and difficulty achieving therapeutic plasma concentrations (2–5.5 mg/L) prompted a switch to olorofim (90 mg twice daily) as salvage therapy. Nine months later, persistent arthritis and hypermetabolic PET activity led to repeat arthrotomy and synovectomy, after which therapy was switched back to high-dose voriconazole (400 mg twice daily). Despite trough concentrations >2 mg/L, septic arthritis persisted, and terbinafine (250 mg once daily) was added.

Given persistent infection despite multiple therapies, antifungal concentrations in synovial fluid were further investigated. Plasma and synovial fluid samples were collected to evaluate voriconazole and terbinafine penetration. Voriconazole, terbinafine, and its inactive metabolite *N*-desmethyl-terbinafine were quantified by liquid chromatography and mass spectrometry. A linear response from synovial fluid serially diluted in drug-free plasma confirmed the suitability of plasma-based calibration for quantification in both matrices. Sampling occurred 15 days after voriconazole monotherapy and 2 days after combination therapy (Table 1).

**TABLE 1:** Antifungal concentrations in plasma and synovial fluid after systemic administration^*a*^

Drug	Days since terbinafine initiation	Oral dosage	Hours since last dose	Plasma (mg/L)	Synovial (mg/L)	Synovial/plasma ratio
Voriconazole^*a*^	−7	400 mg q12h	3	6.32	1.84	0.29
Voriconazole	+2	400 mg q12h	9	4.04	3.26	0.81
Terbinafine	+2	250 mg q24h	9	0.2588	0.1243	0.48
*N*-Desmethyl-terbinafine	+2	N/A	N/A	0.1074	0.0325	0.30

^*a*^ Concentration measured 15 days after the initiation of oral voriconazole monotherapy. q12h: every 12 h, q24h: every 24 h. N/A, not applicable.

Four months after initiation, combination therapy led to marked clinical and radiological improvement. Voriconazole showed substantial synovial penetration (30–80%), with concentrations 3.7–6.5 fold above the MIC, consistent with published human clinical and animal data (3, 4). Synovial concentrations varied despite stable inflammatory markers and albumin. Terbinafine plasma concentrations were higher, whereas metabolite levels were lower, than previously reported (5), with approximately 50% synovial penetration. Although the MIC was >8 mg/L, previously described synergy between voriconazole and terbinafine in *Scedosporium* spp. and *Lomentospora prolificans* may have contributed to the clinical response (6, 7). These findings are consistent with the limited human data summarized by Gamaletsou *et al*. and support adequate penetration of both voriconazole and terbinafine into synovial fluid (8). Although synovial fluid concentrations of olorofim were not available, trough plasma concentrations reached 0.586 mg/L after several months of therapy, corresponding to 39-fold above the MIC and consistent with concentrations reported in clinical trials (9). Data regarding olorofim distribution into bone and joint tissues remain scarce. In a quantitative whole-body autoradiography study in 24 rats receiving a single 2 h infusion of [14C]-olorofim, drug concentrations at the bone surface were lower than those observed in most other tissues, suggesting limited penetration into cortical bone (10). Consistent with these pharmacokinetic observations, a phase IIb study of immunocompromised patients with invasive fungal infections reported relatively low response rates among patients with bone infections, with MSG-EORTC global response success rates of 37.0% (95% confidence interval [CI], 19.4–57.6) at day 42 and 18.5% (95% CI, 6.3–38.1) at day 84 (9). Nevertheless, successful use of olorofim as salvage therapy has been reported in refractory osteoarticular infections, including native knee arthritis due to *Scedosporium apiospermum*, and septic arthritis with osteomyelitis due to *L. prolificans* in immunocompetent patients (11, 12).

Overall, this case highlights the limited data available on antifungal pharmacokinetics in osteoarticular fungal infections. High plasma exposure to olorofim was not associated with clinical improvement, whereas combination therapy with voriconazole and terbinafine achieved measurable synovial concentrations and was associated with a favorable outcome. Further studies are needed to better define intra-articular antifungal pharmacokinetics.
